# Pancreatic 3D Organoids and Microfluidic Systems—Applicability and Utilization in Surgery: A Literature Review

**DOI:** 10.3390/medicina61040623

**Published:** 2025-03-28

**Authors:** Vidas Petrauskas, Ryte Damaseviciute, Aiste Gulla

**Affiliations:** 1 Institute of Clinical Medicine, Faculty of Medicine, Vilnius University, LT-01131 Vilnius, Lithuania; 2 Center of Visceral Medicine and Translational Research, Faculty of Medicine, Vilnius University, LT-01131 Vilnius, Lithuania; 3 Department of Surgery, George Washington University, Washington, DC 20052, USA

**Keywords:** pancreatic organoids, organoid, organ-on-a-chip, PDAC

## Abstract

*Background*: Pancreatic organoids are a rapidly advancing field of research with new discoveries being made every day. A literature review was performed to answer the question of how relevant 3D pancreatic organoids are for surgery. *Materials and Methods*: We started our investigation by identifying articles in PubMed within the last 5 years using the keywords ((“pancreatic organoid”, OR “organ-on-a-chip”, OR “pancreatic chip” OR “3D culture methods”) AND pancreatic surgery). Only English articles were included in this literature review. This literature review was performed in a non-systematic way; articles were chosen without a predetermined protocol of inclusion and were based on the aim of the review. *Results and Conclusions*: There are many promising innovations in the field of 3D cultures. Drug sensitivity testing in particular holds great potential for surgical application. For locally advanced PDAC, EUS-FNB obtained cancer tissue can be cultured as organoids, and after 4 weeks, neoadjuvant treatment could be adjusted for each patient individually. Utilizing this approach could increase the number of R0 resections and possibly cure the disease. Furthermore, microfluidic devices, as a platform for pancreatic islet pre-transplant evaluation or cultivation of *beta* cells derived from HiPSC in vitro, promise broad application of islet transplantation to T1DM patients in the near future.

## 1. Introduction

The pancreas serves as an exocrine and endocrine organ. Pancreatic ductal adenocarcinoma (PDAC) develops from the exocrine part of the gland and is one of the deadliest types of cancer. It accounts for about 93% of all malignancies arising from the pancreas [1,2]. Every year more than 510,000 new cases of pancreatic cancer are diagnosed worldwide, and 467,000 deaths are reported [3]. Although a rather uncommon type of cancer, PDACs prevalence increases by 0.5% to 1.0% annually. Regardless of the systemic treatment advances in the last decades, the 5-year survival rate is still below 10% [4]. The majority of patients diagnosed with pancreatic cancer for the first time have disseminated disease with a mean survival of only 8–11 months despite aggressive chemotherapy [5,6]. Combining surgery with chemotherapy is the only curative option in localized PDAC and increases the 5-year survival to 20–25% [7]. Despite a high number of new drugs in preclinical trials, the molecular heterogeneity and mutations among PDAC limit their effectiveness [8]. To identify which systemic therapy could be the most beneficial to every individual, a more personalized approach and accurate biomarkers are needed.

In recent years, research involving patient-derived 3D organoids has made significant advancements [9,10,11]. Patient-derived cancer organoids (PDO’s) can be cultured ex vivo through multiple passages, allowing enough biomass of the tumor to study the genetic alterations of cancer clone’s DNA and sensitivity to clinically most relevant combinations of chemotherapy [12,13,14]. Originally, organoids were characterized as differentiated progenitor cells (stem cells) with the structure and function matching the tissue of interest for research purposes [15]. The more clinically relevant method is culturing pancreatic tumor organoids obtained from surgical specimens or from endoscopic ultrasound fine needle aspiration or biopsy (EUS-FNA/FNB) [16,17,18,19]. However, since pancreatic organoids after a few passages are composed only of epithelial cells, they lack the tumor microenvironment (TME) in vivo, which is primarily composed of fibroblasts and immune cells and plays a vital role in tumor biology [20]. Therefore, some groups utilize microfluidic devices to co-culture PDAC organoids with mesenchymal or immune cells to study carcinogenesis processes in vitro [21].

If the critical volume of endocrine *beta* cells is damaged by autoimmune disease, T1DM follows [22]. Traditional treatment includes daily insulin injections, continuous glucose monitoring, and hybrid closed-loop systems [23]. Despite this advanced treatment, some patients still suffer from constant hyperglycemia or severe, life-threatening hypoglycemia episodes. *Beta* cell replacement is an option in this situation. Although pancreas transplantation provides long-term normoglycemia, this procedure is associated with significant morbidity and mortality [24]. Islet transplantation is a less invasive option [25]. To add more, islet autotransplantation has improved outcomes in glycemia control after total pancreatectomy due to chronic pancreatitis [26]. We cannot offer islet transplantation for every patient with indications due to a shortage of donors. Therefore, new technologies are implemented for islet research in vitro, including microfluidic devices [27]. A new possible source of *beta* cells—embryonic stem cells (ESC) and human-induced pluripotent stem cells (HiPSC)—poses a great potential to solve the *beta* cell shortage problem [28]. Microfluidic devices can be utilized to create the same size HiPSC-derived *beta* cell organoids (pseudoislets) in a scalable manufacturing manner [29,30].

Within this review, the current literature is analyzed regarding pancreatic organoids and microfluidic systems’ utilization in surgery.

## 2. Materials and Methods

A literature search in the Medline database was conducted to identify relevant articles from the past 5 years using the keywords ((“pancreatic organoid”, OR “organ-on-a-chip”, OR “pancreatic chip” OR “3D culture methods”) AND pancreatic surgery). Only English articles were included in this literature review, in a non-systematic way.

The focus of this review was on these questions: (i) what are the main advantages of organoids?; (ii) how can we implement pancreatic 3D organoids in surgery?; and (iii) microfluidic systems as a platform for pancreatic organoid applications.

## 3. Discussion

### 3.1. Culture Types

Since the first cell culture was established by Harrison in 1907 [31], their development has progressed steadily. This enabled the proper investigation of the mechanisms of formation and function and the pathology of organs and their tissues. Until today, 2D cell cultures are the most common cell culture method being used. In a 2D cell culture system, the cells are grown as a monolayer in a culture flask or flat dish embedded into extracellular matrix (ECM) [32]. Furthermore, there is the suspension cell culture method, in which single cells or cell aggregates multiply in a nutrient fluid until further processing takes place [32]. They do not represent the normal in vivo cell environment since they do not mimic the natural structures of tissues found in the body. Therefore, cell-to-cell and cell-to-ECM interactions are not comparable to those occurring in vivo. Because of this circumstance, the predictive value of 2D culture is limited for drug discovery, testing, or research outcome [33]. Another disadvantage is the unlimited access to all ECM ingredients, which is not representative for tumor cells, i.e., in vivo cancer cells have variable access to these ingredients due to the architecture of the tumor [34]. Based on all these concerning disadvantages, there is a necessity for alternative culturing methods that would be more feasible, mimicking the ECM.

3D cell culture is the next step in cancer research [35,36]. It allows cells to grow in any direction and interact with the ECM or other aggregate cells, resulting in growth that is not limited to the 2D culture medium structure of classical cultures [37]. The main advantages of 3D cultures are that they form complex systems and show significantly better cell-to-cell and cell-to-ECM interaction while also creating “niches”. Cells can obtain signals from the surrounding environment, as it also happens in vivo. Another improvement is the maintenance of characteristics of the cell [38]. In a 3D environment, the cell can retain its unique morphology and mode of division, leading to a more viable organoid. These exclusive properties allow a more intense study of healthy tissue-derived cultures for regenerative medicine, cancer research and drug testing, or testing of the effectiveness of cell response to radiation.

In addition to that, 3D cell cultures can reduce utilization of animal testing in research and are therefore more ethical. [34,39].

There are several types of 3D cultures [40]. The simplest are aggregates or spheroids, which are mainly a collection of cells usually obtained from cell line monocultures [41]. Spheroids are described as 3D cell spontaneous aggregates that do not have contact with the culture substrate (ECM), which results in cell–cell interaction without cell/matrix interaction [42]. Two main culturing methods are utilized in spheroid creation: hanging-drop and ultra-low attachment cultures [43]. Although there is no pre-defined ECM in a spheroid culture to adhere to, it is observed that cells themselves secrete ECM molecules (for example, tripeptide Arg-Gly-Asp) to aid in cell aggregation and a stable structure of a 3D sphere [41].

The next step in 3D cell cultivation is organoids [15,44,45]. According to some authors, there is a distinction among the terms “spheroid” and “organoid” [46]. Spheroid is only a method of 3D cell culturing, while organoids must meet specific requirements to be called so. According to consensus by Marsee et al., an organoid is defined as a “three-dimensional structure derived from (pluripotent) stem cells, progenitor, and/or differentiated cells that self-organize through cell–cell and cell–matrix interactions to recapitulate aspects of the native tissue architecture and function in vitro” [46]. A spheroid can be called an organoid only if it is composed of organ-specific cells and recapitulates the original structure and function of the organ it is mimicking in vitro.

Organoids were originally derived from stem cells; however, during the past decade it has been observed that differentiated cells (for example, primary patient tissue or tumor cells) can be successfully cultured and passaged to study in vitro as well [47]. It is possible to produce a variety of organoids mimicking different organ structures and functions in vitro, including small and large intestine [48], pancreas [49], kidney [50], brain [51], and liver [52]. In contrast to spheroids, organoids can be propagated for a long-term ex vivo study [53]. Although cultivating organoids offers great potential, it is not an easy task [54]. Organoids can be derived from a variety of cells, but they must be carefully isolated and supplied with a specific culture medium including nutrients and growth factors. If embryonic stem cells are utilized, they are first proliferated, and once the predefined number of cells is reached, they are subsequently differentiated in a multistep protocol to mature functional organoids [55].

**Figure 1:**
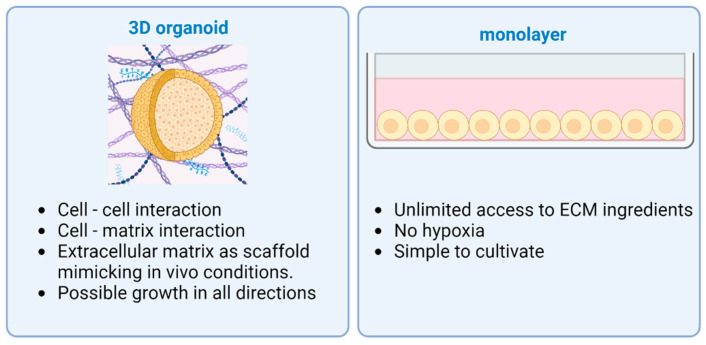
Advantages of different cell culturing methods: 3D organoid vs. 2D monolayer. Two-dimensional monolayers of cells have unlimited access to ECM ingredients, suffer less from hypoxia, and are simple to cultivate. Three-dimensional organoids are more complex structures with cell–cell and cell–matrix interaction, as well as possible growth in every direction. Created with BioRender.com.

ECM is essential to maintain a niche for the stem cells during differentiation with physical and chemical stimulation [56,57]. Together with natural ECM, the most widespread type is Matrigel purified from Engelbreth-Holm Swarm mouse sarcoma [39]. Specific growth factors and differentiation modulators are added to the growth media for differentiation and controlled growth [58]. Also, organoids can be established from induced pluripotent stem cells (iPSCs) or adult stem cells (ASCs) taken from normal tissue. Tissue-derived stem cells are able to reorganize themselves into organoids, mostly composed of epithelial cells [59]. The cell source, the culturing media, and the ECM should be chosen according to the organ and the result that is intended to be achieved. Compared to other organs such as the liver, the pancreas in vivo has a very limited ability to regenerate, neither in homeostasis nor when injured. This circumstance complicates the production of pancreatic organoids [60], which is why iPSCs are often used to achieve good results. Organoids differentiated from iPSCs are complex structures and pose a possibility to include multiple germ layer cell types: endothelial, epithelial, or mesenchymal cells. Supporting cells in complex pancreatic organoids usually result in enhanced viability and function in vitro [61]. The main differences between 2D and 3D cultures are provided in Figure 1.

Even though conventional organoid types are very similar to in vivo organs, it remains difficult to precisely control their development. Another limitation is the inability of conventional organoids to provide a complex micro-environment. For this reason, microfluidic systems were implemented in cell culturing and provide controllable experimental conditions for the organoid [62]. This combination of 3D cell culturing and microfluidic devices is called organ-on-a-chip (OOAC) [63]. As described by Qirui Wu et al. [64], OOAC cultures have the ability to mimic the organ much better than other culture types. By adding a microfluidic channel network, it is possible to influence important parameters of the culture such as concentration gradient and tissue-organ interactions. The design is different depending on the organ studied, but every device contains chambers for cells to grow while constantly being perfused by culture media [65]. It differs from classical organoid cultures on Matrigel, where culture medium is changed only every 5 days [66].

In vivo epithelial cells are always supported and interacting with surrounding tissues, including endothelium, mesenchymal, or immune cells [67]. These are essential elements to replicate in vitro physiological environment if tumorigenesis is studied, for example, cancer potential to metastasize or immune cell-induced tumor necrosis. The multi-organ-on-a-chip model provides great possibilities. However, the complexity of the system results in reduced reproducibility and challenges to be solved in the future [68].

### 3.2. PDAC Organoid Clinical Applications

#### 3.2.1. Drug Sensitivity Testing

Pancreatic cancer, and especially PDAC, has high heterogeneity, and multiple cancer cell clones cause the aggressive systemic treatment to be ineffective with many side effects [69]. Nowadays, two main chemotherapy schemes are used to treat disseminated PDAC. Gemcitabine/nab paclitaxel (G/A) compared to FOLFIRINOX (5-fluorouracil, leucovorin, irinotecan, and oxaliplatin) is a less toxic combination but provides inferior efficacy [70]. Furthermore, if the patient received neoadjuvant chemotherapy, organoids derived from surgical specimens usually manifest with resistance to previous therapy due to emerging new PDAC clones and mutations [71,72].

PDAC is already a disseminated disease in 75–80% of patients; therefore, chemotherapy is the only possible treatment option to slow down the tumor progression and prolong survival for a few months [70]. Since PDAC is a very fast-growing tumor, speed is of utmost importance, especially for borderline tumors for which surgical resection is possible. Nowadays, we do not have sensitive and specific biomarkers to guide clinical decision-making to choose the most effective chemotherapy regimen for the individual patient. Historically, patient-derived xenografts (PDX) in vivo were the gold standard in drug sensitivity testing for PDAC [67]. However, this is a laborious and time-consuming method and lasts 6–8 months, which is clinically irrelevant [69]. To overcome this limitation, patient-derived organoids (PDO) were established [71,73]

Organoid cultures can be utilized even in predicting treatment response [74]. Organoid technology can recapitulate the original tumor genetics and structure with astonishing precision, not inferior to PDX. A study carried out by Frappart et al. [75] compared PDX organoids with patient-derived organoids with a small-scale sensitivity test. The authors concluded that organoids demonstrate the same feasibility for sensitivity testing as PDXs. In addition, organoids have higher predictive value because they do not have unwanted interactions between xenografts and animal cells.

Hervé Tiriac et al. [76] generated a patient-derived organoid library that is able to summarize the mutational spectrum and transcriptional subtypes of primary pancreatic cancer. Furthermore, they defined new oncogenes, and continued analysis showed unique clusters, which could be used to improve treatment. A case study was performed that predicted improved response for patients due to organoid-based gene expression signatures. Finally, Tiriac et al. propose that it is possible to predict clinical response by combined molecular and therapeutic profiling, which could result in a more accurate therapy. Other research groups have also come to similar results. It is possible to find concordance between patient-derived organoids and thus enable a more targeted therapy [77].

Organoid development consists of three phases: establishment, expansion, and characterization [69]. Dantes et al. report that a small biomass of organoids can be tested just after the organoid establishment phase. The supernatant is collected, and cell-free DNA is analyzed to guide specific immunotherapy in just 2 weeks after sample collection [78]. Further pharmacotyping requires larger biomass, so the expansion phase has to be completed; usually it lasts 4 weeks [69]. Success of organoid cultures obtained from surgical specimens or EUS-FNB to at least five passages and subsequent pharmacotyping in the leading world laboratories reaches over 80% [13,19].

It is even possible to establish organoid cultures non-invasively from circulating tumor cells obtained from a patient’s blood sample. Wu and colleagues reported culture efficiency of 87% in just 3 weeks of cultivation [79].

For patients who went through surgery, effective postoperative chemotherapy is an important adjunct to increase survival [80]. Seppälä et al. [81] established patient-derived organoids from surgical specimens and endoscopic biopsies for high-throughput drug testing to explore if drug sensitivity testing could be performed within a clinically meaningful timeframe. Single cells were placed in liquid Matrigel and supplied with culture medium. For sensitivity testing, single cells were placed on a 384-well assay plate in 10% Matrigel and tested for different chemotherapeutics. It was possible to obtain results rapidly within 18 days with a mean time of 49 days. The median time between surgery and initiation of chemotherapy was set to be 62 days according to a pancreatectomy database survey. This and other studies show that there is also a possibility to utilize organoids in the postoperative phase and that the results obtained can be used to choose the potent chemotherapy [82,83].

Despite the advantages of PDOs, there are still many challenges remaining for this technology to be an important part of clinical decision-making [69]. First of all, PDAC is a paucicellular tumor—the majority of the specimen consists of stromal cells, and only 15% of epithelial tumor cells [84]. A meta-analysis recently conducted by Grützmeier et al. reported that actual organoid procurement success with pharmacotyping is 60% for EUS-FNB, 36% for percutaneous biopsies, and 62% for surgical specimens [85]. Many patients are diagnosed with advanced disease, and sometimes they receive neoadjuvant systemic treatment prior to organoid establishment. It is observed that neoadjuvant chemotherapy and radiotherapy reduce chances to culture PDOs substantially [86]. This real-world data suggests that one-third of patients will not receive personalized treatment for PDAC. Unfortunately, specific immunotherapy today is applied only to a few percent of PDAC patients [69]. To add more, during PDO’s culture expansion, the stromal compartment is lost during the first two passages due to specific growth factors in the culture medium supporting only epithelial cell proliferation. Consequently, tumor microenvironment (TME) is lost, and PDOs no longer mimic in vivo cancer biology. Another interesting phenomenon was observed during the expansion phase of organoids. 

Before the first passage, the most abundant genetic clone of PDAC usually is not the same after 5 passages [81]. The dominant clone requires time to overwhelm the remaining tumor variants. This means that after the last PDO’s passage, specific systemic treatment is directed only against one variant of the tumor, ignoring the rest. Finally, there are still no RCTs conducted to confirm PDO’s real impact in clinical setting.

Microfluidic technology provides some solutions for PDO’s culturing limitations. To begin with, a continuous flow of fresh culture medium provides essential growth factors and oxygen to organoids and prolongs viability. Also, microfluidic system design aids in uniform-size PDOs compared to 3D culturing methods on Matrigel [9].

Zhang et al. [87] introduced a scalable multiplexed drug-combination screening platform which uses 3D microtumor models. They produced a chip with a “Christmas tree mixer” structure with the ability to provide a large drug concentration range for screening. This high-throughput combination screening scheme has the potential to test nearly any number of drugs and their pairwise combination with multiple logarithmic mixing ratios. To test for efficacy and applicability of this chip, a 8-drug combination chip was implemented. The drug combination screening was performed with breast and pancreatic cancer cell lines [88]. For pancreatic testing MIA PaCa-2 cell lines were used. Seven chemo-drugs and one media only as a positive control were screened, resulting in 172 different treatment settings. While testing cisplatin, docetaxel, doxorubicin, gemcitabine, irinotecan, oxaliplatin and fluorouracil as a single drug or in different combinations, they saw a few treatment combinations which showed higher effectiveness compared to single drug therapy. Even though this study was based on MIA PaCa-2 cell lines, the relevance of OOAC can be inferred i.e., the possibility to test not only single drugs, but even interactions of several drugs, shows great potential.

One of the main advantages of OOAC is co-culturing PDAC organoids with essential TME cells, for example stellate cells or lymphocytes [89,90]. These interactions observed iv vitro could lead to specific stroma or immune system targeting therapy.

Davenport et al. [91] co-cultured multiple cell types (patient-derived pancreatic organoids, human fibroblasts, and endothelial cells), placed on a flow controlled OOAC platform and tested drug sensitivity. Their results demonstrate that the value of a perfusable vascular network showed better reaction for drug screening compared to stiffened matrix.

#### 3.2.2. Regenerative Medicine

Another area of research which is getting more attention is the study of diabetes mellitus (DM). Mortality rates in lower-middle-income countries increased by 13% between 2000 and 2019 [92]. Therefore, sufficient treatment is more important than ever. The idea of culturing pancreatic islets for transplantation has been around for a while. However, there have been some problems in producing these islets, i.e., they are more dependent on external factors, such as blood flow and interactions between cells, than other cell structures. Without these factors, cells are unable to maintain a good blood-glucose homeostasis. The introduction of 3D and OOAC models solved some of these issues. A comparative study between different types of spheroid production demonstrated that islet cell spheroids, generated either in locally fabricated silicon microwells or in Sphericalplate 5D, yielded highly functional insulin-producing constructs with minimal labor [93]. Although providing better results than previous culturing methods, spheroids have limited application abilities. Due to their structure, spheroids are very susceptible to oxygen. When they reach a certain size (approximately 150 µm in diameter), it is no longer possible to achieve sufficient oxygenation of the inner cells leading to necrosis, limiting the size and usability of such cultures.

Pancreas transplantation is an option to treat T1DM in the long term. However, it is a major surgical procedure with high morbidity and mortality [94]. Pancreatic islet transplantation is therefore a good alternative with lower morbidity [95]. For islet transplantation several OOAC models have been designed, which could bring further prospects [96]. Culturing pancreatic islets on microfluidic chips involves trapping sides, mostly micro-wells, where islets are immobilized and cultured under continuous flow [97]. It has been shown that the size of the islets should be as consistent as possible, where smaller islets are more viable and produce more insulin if normalized for their mass than the larger ones [98]. Nowadays hiPSC differentiation protocols produce viable and functional *beta* cells in vitro comparable to native islets [99]. Furthermore, there are promising bioengineering solutions for massive islet production such as microwells (with centrifugation) [29] and hanging drop systems [30]. Before transplantation of the islets, they need to be tested for purity, function and viability. Enzyme-linked immunosorbent assay (ELISA) is still the predominant method used, which requires the separation of the cells, making this method very time and cost-consuming. On-chip testing could therefore save a lot of resources. Hori et al. [100] developed a compact fluidic system for the assessment of islet functionality called g-STAR. This novel system uses a micromesh sheet-embedded chip. Islets which are easily placed on the mesh are trapped and held in place. Culture medium was pumped through the chip. The system was complemented by a sample fraction chip for fluid collection. The functional test with high and low glucose levels was performed with murine pancreatic islets to test the performance of the system. The results have indicated that the system successfully analyzes insulin secretion, which confirms that it is able to evaluate the quality of islets cheaply and quickly. Thanks to improved islet cell assessment methods, pancreatic islet transplantation is becoming safer and more successful.

### 3.3. Current Trends and Future Directions

**Figure 2:**
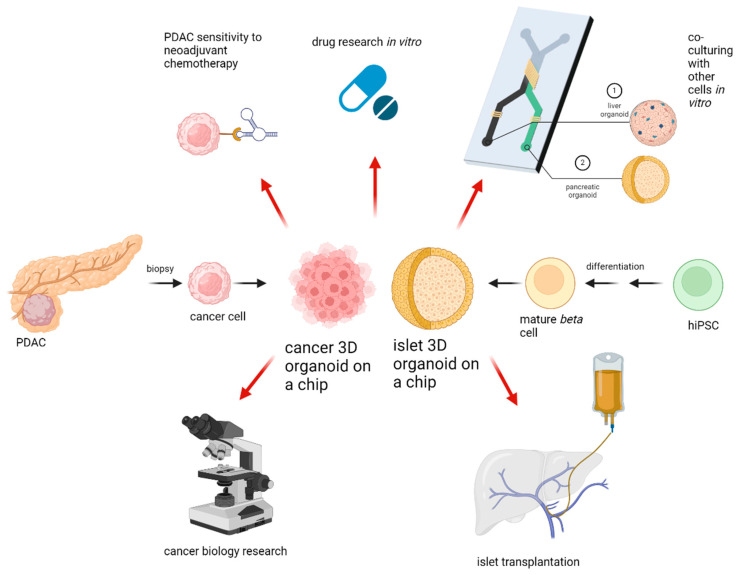
Applications for 3D organoids. After PDAC biopsy, 3D cultures of cancer cells can be established. If pharmacotyping is successful, personalized chemotherapy or immunotherapy can be initiated. HiPSC can be differentiated to mature *beta* cells followed by same-size organoid formation in microfluidic devices with subsequent islet transplantation into the portal vein. Pancreatic cancer or islet organoids can be co-cultured with other cells in vitro to create a multiple organ-on-a-chip model for further drug or cell biology research. Created with BioRender.com.

Three-dimentional culture systems have been under research for quite some time, with new systems being released and new fields of applicability can be seen everywhere. With increasing cellular complexity, culture systems are more error-prone and less reproducible than traditional cultures. The in-troduction of OOAC devices increased the effectiveness of organoids by combining microfluidic systems and organoid cultures. The key directions for organoid applicability in research and clinical setting are provided in Figure 2. What is important to remember is that any culturing device needs to be appropriate for its specific purpose, since every organ needs its unique setup. Until now there is no device which can replace animal testing totally. Another drawback of organoid systems is the lack of interorgan communication. However, research is currently underway to address these challenges as well, and promising novel multi-organ-on-a-chip devises will represent the human body even more accurately in the future than previous systems [47]. In the treatment of pancreatic tumors, a great challenge lies within the low resectability rates. Organoids can reduce this problem through high-throughput sensitivity testing methods, enabling a more individualized medication. Even though PDAC has high heterogeneity, organoid sensitivity testing can hopefully lead to successful treatment results, subsequently resulting in tumor shrinkage. With the implementation of rapidly evolving technology, an unresectable PDAC might be curable in the near future. To add more, in the field of pancreatic islet transplantation, organoids could be used with great advantage for production and evaluation of islets. In particular, using OOAC devices, it is possible to produce small size islets in large numbers, making islet transplantation utilizing pluripotent stem cells a relevant alternative to pancreas transplantation. We confirmed the great advantages and potential which 3D culture systems hold. Further research has to demonstrate whether these advantages can also be applied in clinical practice.

## 4. Conclusions

Despite difficulties in 3D organoid cultures or microfluidic systems, these technological advances will alter management of pancreatic diseases. If the majority of patients with borderline resectable PDAC could be treated with personalized neoadjuvant chemotherapy or immunotherapy, more patients would benefit from R0 resection. To add more, microfluidic device utilization in proliferating, differentiating, and preparing same-size pseudoislets derived from HiPSC would increase islet transplantation numbers substantially. Further research in these technologies is necessary to make a significant impact in pancreatic surgery.

## Data Availability

No new data were created or analyzed in this study. Data sharing is not applicable to this article.

## Abbreviations

The following abbreviations are used in this manuscript:

ASC	Adult stem cells
DM	Diabetes mellitus
ECM	Extracellular matrix
ELISA	Enzyme-linked immunosorbent assay
ESC	Embryonic stem cells
HiPSC	Human induced pluripotent stem cells
OOAC	Organ-on-a-chip
PDAC	Pancreatic ductal adenocarcinoma
T1DM	Type 1 diabetes mellitus
TME	Tumor microenvironment
